# Myeloid-derived suppressor cell (MDSC)-like neutrophils induced by pulmonary infection with *Coccidioides posadasii* exacerbate disease by suppressing CD4^+^ T cell immunity

**DOI:** 10.1128/mbio.00772-26

**Published:** 2026-05-28

**Authors:** Nawal Abdul-Baki, Austin Negron, Reimi Navarro, Althea Campuzano, Matthew Mendoza Barker, Nicolas D. Prather, Paul L. Fidel, Thomas G. Forsthuber, Jieh-Juen Yu, Chiung-Yu Hung

**Affiliations:** 1 Department of Molecular Microbiology and Immunology, South Texas Center for Emerging Infectious Diseases, The University of Texas at San Antonio 843937, San Antonio, Texas, USA; 2 Center of Excellence in Oral and Craniofacial Biology, School of Dentistry, Louisiana State University Health Sciences Center 12258, New Orleans, Louisiana, USA; Texas Christian University, Fort Worth, Texas, USA

**Keywords:** *Coccidioides*, coccidioidomycosis, Valley fever, myeloid-derived suppressor cells, T cell proliferation

## Abstract

Coccidioidomycosis is a pulmonary disease caused by the dimorphic fungus *Coccidioides*, which is endemic to the southwestern United States and South America. *Coccidioides* grows as septate hyphae in soil and forms spores that can become airborne during mechanical disruption of the soil. In the lungs, the inhaled spores develop into large multinucleated spherules, a unique morphology among medically important *Coccidioides* species, and reproduce by endosporulation. A hallmark of the host immune response toward endosporulating spherules is the robust recruitment and infiltration of Ly6G^+^ neutrophils. However, the effectiveness of Ly6G^+^ neutrophils against *Coccidioides* remains to be studied. Myeloid-derived suppressor cells (MDSCs) are immunosuppressive and share an origin and many phenotypic features with neutrophils. Here, we demonstrated that Ly6G^+^ cells, but not Ly6C^+^ cells, increased lung expression of the inhibitory programmed death-ligand 1 (PD-L1) in diseased mice. The Ly6G^+^PD-L1^+^ cells share common characteristics of granulocytic MDSCs. Bone-marrow-derived GR-1^+^ myeloid cells, including both Ly6G^+^ and Ly6C^+^ cells, suppressed the proliferation of CD4^+^ T cells after exposure to spherules. Furthermore, isolated GR-1^+^ cells from the lungs and spleens of *Coccidioides*-infected non-protected mice also suppressed CD4^+^ T cell proliferation, thereby diminishing adaptive T cell responses. Adoptive transfer of GR-1^+^ myeloid cells isolated from the lungs of *Coccidioides*-infected mice, or derived from bone marrow exposed to paraformaldehyde-killed spherules, into vaccine-protected recipients exacerbated coccidioidomycosis through the reduction of protective Th1 and Th17 responses. Altogether, we demonstrate that pulmonary infiltration of MDSC-like neutrophils, in non-vaccinated mice, play a detrimental role in coccidioidomycosis by inhibiting protective CD4^+^ T cell responses.

**IMPORTANCE:**

Dimorphic fungi are primary pathogens that infect both immunocompetent and immunocompromised hosts. Fungal pathogens have evolved sophisticated strategies to evade innate immune responses, which have not been well studied. Recently, myeloid-derived suppressor cells (MDSCs) have been described in the pathogenesis of several fungal infections, including *Candida albicans* and *Cryptococcus neoformans*. Here, we use a live-attenuated vaccine model to investigate the role of Ly6G^+^ myeloid cells during *Coccidioides* infection in mice. We have shown that pulmonary *Coccidioides posadasii* infection leads to the accumulation of programmed death-ligand 1 (PD-L1)^+^Ly6G^+^ MDSC-like neutrophils that suppress CD4^+^ T cell proliferation and exacerbate disease by reducing protective Th1 and Th17 responses. Moreover, adoptive transfer of GR-1^+^ myeloid cells isolated from *Coccidioides*-infected mice into vaccine-protected recipient mice reduced protective efficacy by increasing fungal burden and disease severity. Our data suggest that immunosuppressive Ly6G^+^PD-L1^+^ MDSC-like neutrophils play a detrimental role by suppressing protective T cell responses in pulmonary *Coccidioides* infection.

## INTRODUCTION

Coccidioidomycosis (CM) is a fungal pulmonary disease caused by *Coccidioides posadasii* (*Cp*) and *Coccidioides immitis* (*Ci*), which are endemic to the southwestern United States, Mexico, and South America (1, 2). *Coccidioides* species are soil-dwelling fungi that produce arthroconidia, which undergo isotropic growth into parasitic spherules (>40 µm in diameter) in the host lungs (3). Asexual growth of a spherule by endosporulation can produce 300–800 endospores (2–10 µm), which can spread to the skin, bones, and central nervous system, leading to disseminated disease (4). *Coccidioides* species are primary pathogens that can cause symptomatic disease in both healthy and immunocompromised individuals. CM has a mortality rate varying from 5% to 75%, partially influenced by the dissemination location, age, immune status, and other host factors (5–8). Elucidating the immune responses contributing to CM is of utmost importance for understanding the pathogenesis of *Coccidioides* spp.

Mouse and human studies have revealed elevated levels of Ly6G^+^ neutrophils (PMNs) in the lungs, bronchoalveolar lavage fluid, and peripheral blood, a hallmark of CM (9–13). Ly6G^+^ neutrophils isolated from human blood samples can kill coccidioidal arthroconidia and young spherule initials but fail to inhibit the growth of mature spherules (14, 15). In mice, vaccine-induced immunity to *Coccidioides* infection requires Th1 and Th17 memory responses, which in turn promote the infiltration and activation of Ly6G^+^ neutrophils (11, 16). Indeed, depletion of neutrophils in vaccine-protected mice with an anti-Ly6G-specific monoclonal antibody (clone 1A8) impairs protection during pulmonary infection (17). These data insinuate that Ly6G^+^ neutrophils are phenotypically heterogeneous and play multifaceted roles in *Coccidioides* infection. Although the host-pathogen interaction is not yet fully understood, injection of a spherule outer wall fraction, which contains fungal cell wall components such as β-1,3-glucan, chitin, and lipids, reduces the fungicidal capacity of Ly6G^+^ neutrophils *in vitro* and reduces fungal clearance *in vivo* (18). Collectively, these data indicate that Ly6G^+^ cells are functionally heterogeneous cells with canonical pathogen-killing and immune-modulating capacity.

A subset of myeloid-derived suppressor cells (MDSCs), known as granulocytic MDSCs (PMN-MDSCs), express Ly6G and share the same developmental origin as canonical neutrophils (19). PMN-MDSCs and monocytic MDSCs (Ly6C^+^) are two major cell types that deploy several inhibitory mechanisms to suppress T cell function, including expression of inhibitory molecule programmed death-ligand 1 (PD-L1), production of IL-10, nitric oxide (NO), arginase-1, and reactive oxygen species, depending on the immunological context (20–31). While MDSC-mediated T cell suppression has been studied in bacterial, parasitic, and viral infections, the roles of MDSCs in fungal infections have only been investigated for opportunistic infections such as *Candida albicans*, *Cryptococcus neoformans*, and *Paracoccidioides brasiliensis* (32–34) but not in primary dimorphic fungal pathogens. In this study, we hypothesize that the Ly6G^+^ granulocytes that infiltrate the lungs of *Coccidioides-*infected mice, often referred to as neutrophils, are predominantly PD-L1^+^ MDSC-like, which can inhibit CD4^+^ T cell responses and exacerbate disease outcomes. The function of MDSC-like neutrophils was delineated by the adoptive transfer of GR-1^+^ myeloid cells isolated from *Coccidioides*-infected mice into immunoprotected recipient mice immunized with a live-attenuated vaccine. Our data demonstrated that infiltrating MDSC-like neutrophils in non-protected mice play a detrimental role in CM by suppressing protective CD4^+^ T cell responses.

## RESULTS

### Exposure to *Cp* causes an accumulation of Ly6G^+^ granulocytes in non-vaccinated mice

**Fig 1:**
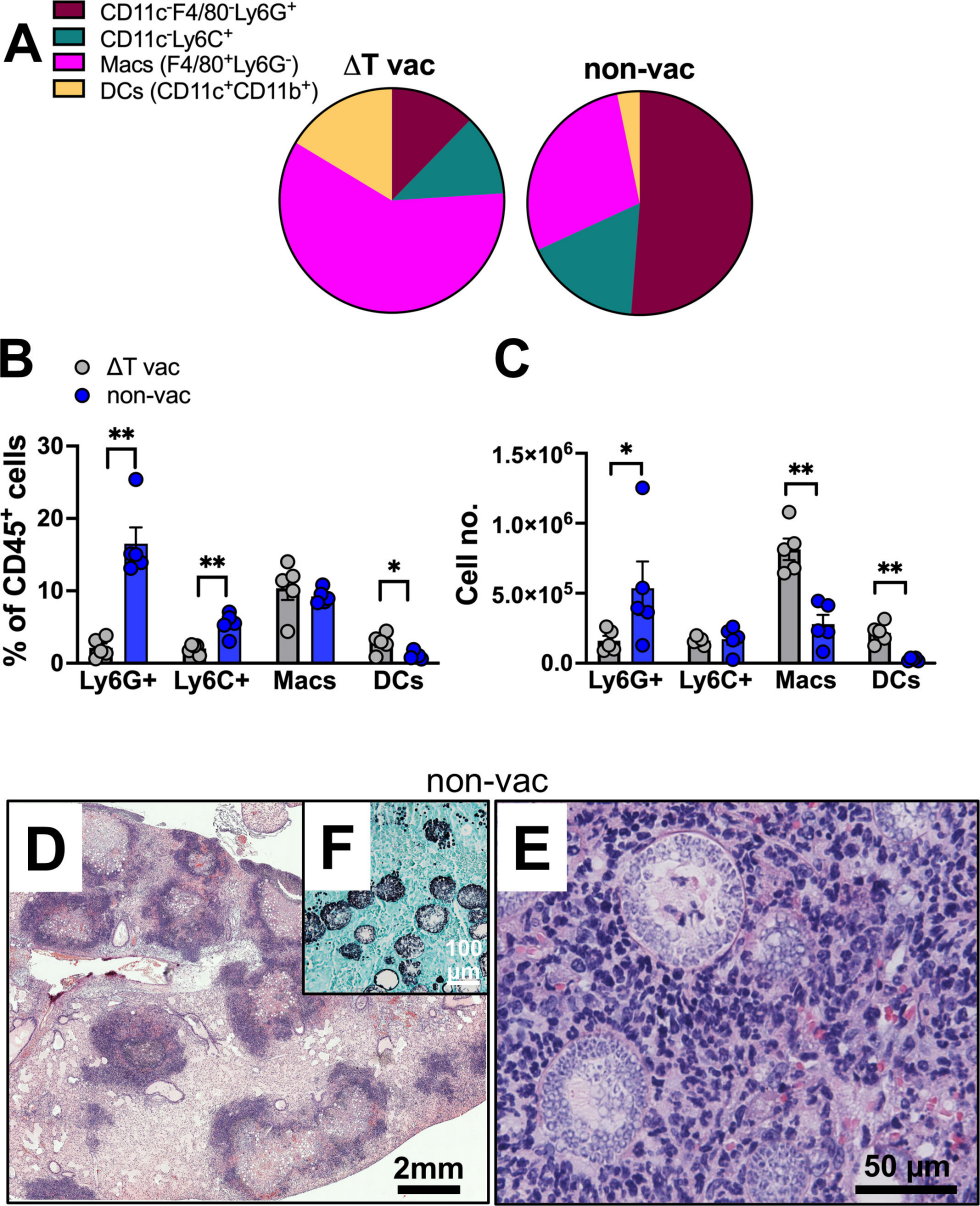
Pulmonary *Coccidioides* infection causes the infiltration of Ly6G^+^ cells into the lungs of non-vaccinated mice. Mice were subcutaneously immunized twice with a live-attenuated ΔT vaccine and intratracheally challenged with a lethal dose of the *Cp* C735 strain. *Cp*-challenged non-vaccinated mice were used as a control. On day 7 post-challenge, the leukocyte population in the lungs was analyzed by flow cytometry. The frequency of Ly6G^+^ cells (CD45^+^CD11c^−^F4/80^−^Ly6G^+^), Ly6C^+^ cells (CD45^+^CD11c^−^Ly6C^+^), macrophages (CD45^+^F4/80^+^Ly6G^−^), and DCs (CD45^+^CD11c^+^CD11b^+^) was calculated (*n* = 5 mice per group; one experiment). The percentages of cells are depicted in a pie chart for the view of cell composition (**A**), and bar charts (mean ± SEM) show cell percentages (**B**) and cell numbers (**C**). On day 14 post-challenge, lungs were collected for histopathologic assessment. Histopathologic sections were prepared using standard hematoxylin and eosin (**D and E**) or Gomori methenamine silver (**F**) staining protocols. Microscopic images at 100× (**D and E**) and 400× (**F**) magnification were collected using an inverted microscope (Leica DMI6000), and mosaic images were generated using Surveyor software (Objective Imaging Ltd.). The microscopic images taken of the lungs from non-vaccinated mice, showed multiple lesion formation (**D**), abundant leukocyte infiltration (**E**), and many detectable GMS-stained spherules (**F**). Data were analyzed by Mann-Whitney to compare innate cell types between ΔT-vaccinated and non-vaccinated mice. *P ≤ 0.05, **P ≤ 0.01.

Flow cytometry analysis of the pulmonary cells prepared from vaccinated and non-vaccinated C57BL/6 mice at 7 days post-challenge (DPC) revealed significant differences in granulocyte accumulation (Fig. 1A through C). Specifically, the percentage and cell number of CD11c^−^F4/80^−^Ly6G^+^ neutrophils (Ly6G^+^) were significantly higher in non-vaccinated mice compared to mice vaccinated with ΔT, an attenuated live vaccine strain of *Cp* (Fig. 1A through C). Moreover, there was no significant difference in the cell number of Ly6C^+^ cells (CD11c^−^CD11b^+^). Conversely, the cell numbers of conventional dendritic cells (DCs, CD11c^+^CD11b^+^) and macrophages (F4/80⁺Ly6G⁻) were significantly upregulated in ΔT-vaccinated mice (Fig. 1A through C). Histopathologic assessment of *Cp*-challenged mice revealed that non-vaccinated mice exhibited multiple lesions indicative of unprotected *Cp* infection in the lungs, with readily visible fungal parasitic cells (Fig. 1D and F). Furthermore, representative hematoxylin and eosin-stained images suggest that the accumulated immune cells in non-vaccinated mice are largely polymorphonuclear granulocytes responding to *Coccidioides* infection (Fig. 1E). Consistent with published data, these findings demonstrate that Ly6G^+^ cells accumulate *in vivo* in non-vaccinated mice in response to *Cp* infection (35, 36).

### Exposure to *Cp in vitro* promoted Ly6G^+^ myeloid cells to upregulate the expression of a co-inhibitory molecule, PD-L1

**Fig 2:**
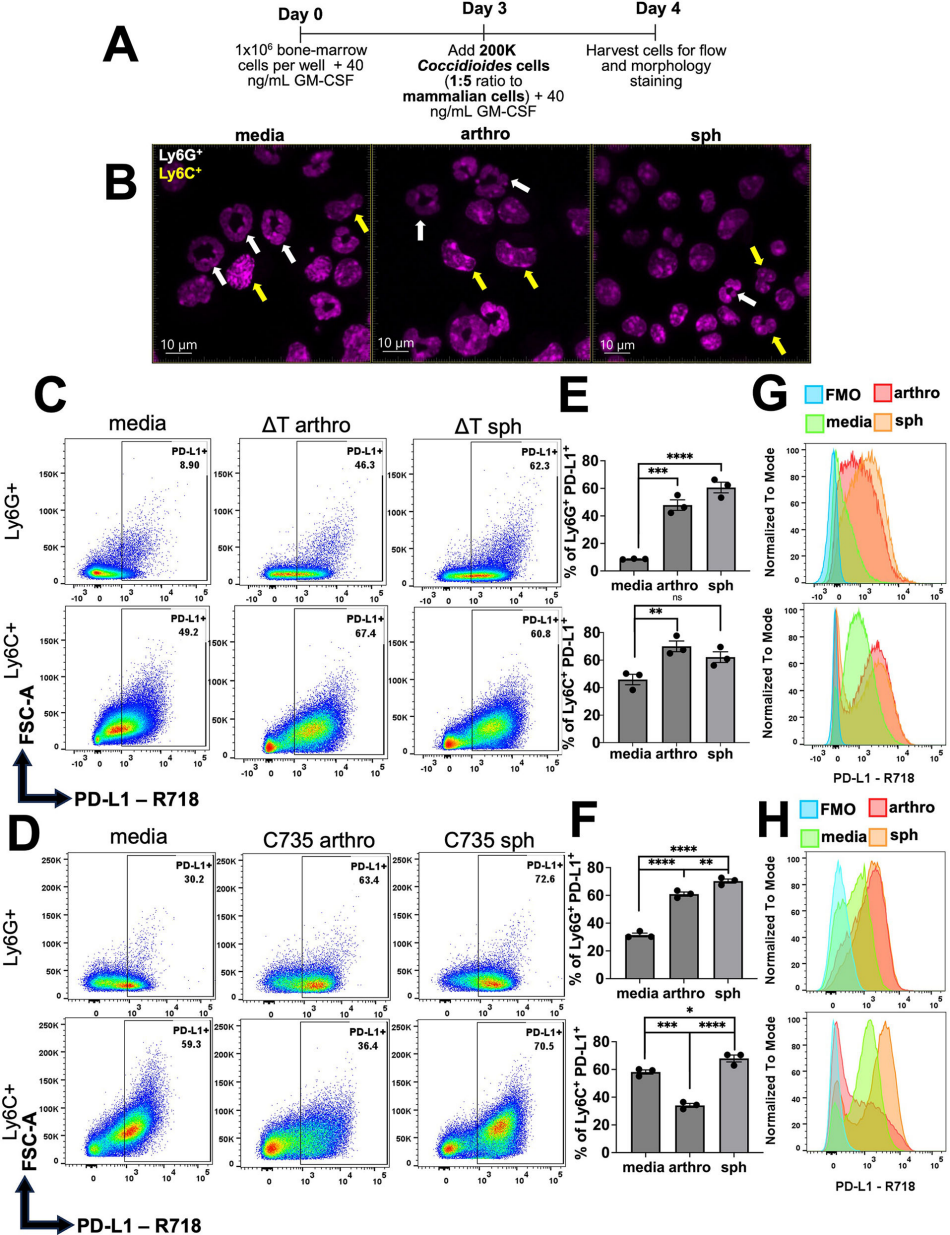
Exposure to arthroconidia and spherules from an attenuated and clinical strain of *Coccidioides* causes CD11b^+^Ly6G^+^ and Ly6C^+^ cells to upregulate inhibitory molecule PD-L1 *in vitro*. (**A**) Generation of bone marrow-derived CD11b^+^Ly6G^+^ and Ly6C^+^ cells exposed to PFA-fixed *Cp* arthroconidia and spherules for 24 h before harvest. (**B**) Representative confocal images of differentiated CD11b^+^Ly6G^+^ and CD11b^+^Ly6C^+^ cells, stained with Hoechst and imaged using the Zeiss 710 NLO 2P System microscope at 100×. Representative plots of gated Ly6G^+^ and Ly6C^+^ cells for their expression of PD-L1 after co-incubation with arthroconidia (arthro) and spherules (sph) isolated from an attenuated mutant (ΔT) and virulent isolate C735 (**C and D**). Data were analyzed using FlowJo 10.9 software, and percentages (**E and F**) and the mean fluorescence intensity of PD-L1 for each gated CD11b^+^Ly6G^+^ and Ly6C^+^ sample were plotted. Fluorescence minus one (FMO) was used as a negative control for PD-L1 gating (**G and H**). Data are mean values ± SEM; *n* = 3 mice per group for flow cytometric analysis; representative of three experiments. *P ≤ 0.05, **P ≤ 0.01: significantly higher percentages of PD-L1^+^ cells in the arthroconidium- and spherule-treated bone-marrow-derived myeloid cells compared with those incubated with medium alone by one-way ANOVA test, respectively.

To investigate the role of these infiltrated granulocytes in non-vaccinated mice, we first assessed GM-CSF-grown bone marrow-derived cells (bone marrow-derived myeloid cells [BMMCs]) for their differentiation and phenotype in the presence of paraformaldehyde (PFA)-killed coccidioidal arthroconidia and spherules. The culture conditions and treatment schedule are outlined in Fig. 2A. Percentages of Ly6G^+^ granulocytes and Ly6C^+^ monocytes were determined by flow cytometry using the gating strategy shown in Fig. S1 to select live, CD45^+^CD11b^+^ myeloid cells, which were then further separated based on Ly6G and Ly6C expression levels. Fixed fungal cells were used in assays to prevent *in vitro* killing of BMMCs by fungi. Cultures were characterized by relatively equal percentages of CD11b^+^Ly6G^+^ granulocytes and CD11b^+^Ly6C^+^ monocytes, as demonstrated by their Hoechst-stained nuclear morphologies (Fig. 2B) and flow data. We further phenotyped BMMCs for their expression levels of the immunosuppressive inhibitory protein PD-L1 after 24 h exposure to either arthroconidia or spherules isolated from the *C. posadasii* ΔT strain (Fig. 2C, E and G) or the clinical virulent isolate *C. posadasii* C735 (Fig. 2D, F and H). The percentages and mean values of fluorescence intensity of PD-L1^+^CD11b^+^Ly6G^+^ cells were significantly higher after exposure to arthroconidia and spherules prepared from both the attenuated strain (ΔT) and the virulent C735 isolate, compared to cells in culture medium alone (Fig. 2C through H). In contrast, the levels of PD-L1 expression on CD11b^+^Ly6C^+^ cells fluctuated after exposure to killed arthroconidia and spherules (Fig. 2C through H). These results suggest that CD11b^+^Ly6G^+^ cells consistently elevated PD-L1 expression upon exposure to *Coccidioides* cells.

### *Cp* infection led to elevated expression of PD-L1 and IL-10 on CD11b^+^Ly6G^+^ cells *in vivo*

**Fig 3:**
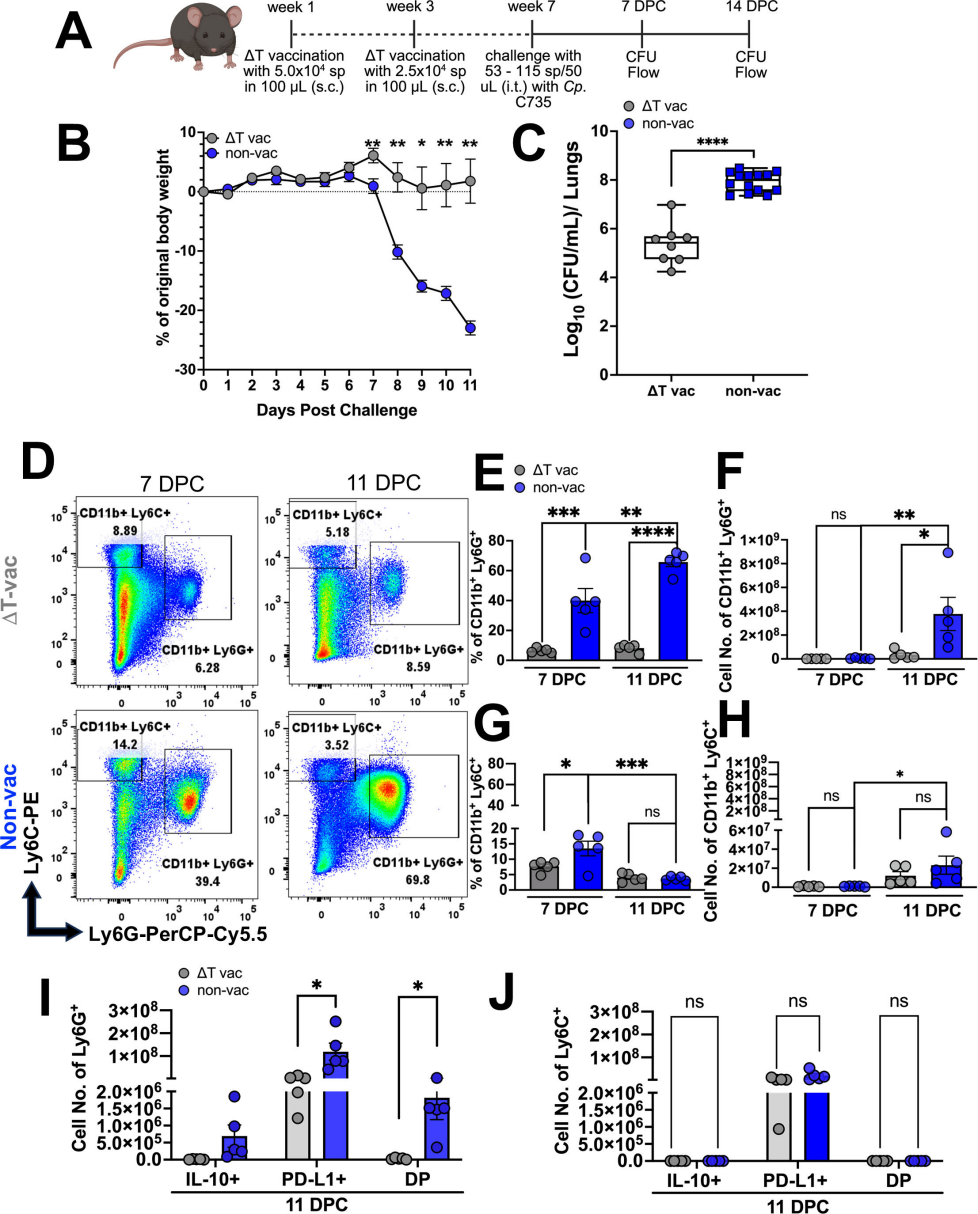
*Coccidioides*-infected mice showed elevated infiltration of CD11b^+^Ly6G^+^ cells, which resulted in elevated expression of PD-L1 and IL-10. (**A**) Schematic illustration of the vaccination and pulmonary challenge protocol for C57BL/6 mice with *Coccidioides* spores. (**B**) The daily bodyweight change (%) in C57BL/6 mice that were subcutaneously vaccinated with an attenuated vaccine strain (ΔT, gray circles) or treated with PBS (non-vaccinated, blue circles) for 11 days after an intratracheal challenge with *Coccidioides* spores. (**C**) Pulmonary fungal burden of vaccinated versus non-vaccinated mice at 11 DPC (**D**) Representative flow cytometry plots of Ly6G and Ly6C fluorescence intensities of the gated, live CD45^+^CD11b^+^CD11c^−^ leukocytes prepared from the lungs of vaccinated and non-vaccinated mice at days 7 and 11 post-challenge. Bar plots of mean + SEM of percentages (**E**) and total cell numbers (**F**) of CD11b^+^Ly6G^+^ cells in the lungs of vaccinated (gray bars) versus non-vaccinated mice (blue bars). Those data for CD11b^+^Ly6C^+^ cells were plotted in panels **G** and **H**. The total numbers of IL-10^+^, PD-L1^+^, and double-positive (DP) cells in the pulmonary CD11b^+^Ly6G^+^ population (**I**) and the CD11b^+^Ly6C^+^ population (**J**) were plotted. *n* = 5 mice per group for flow cytometric analysis; representative of two experiments. The Mann-Whitney *U* test was used to compare body weight and CFU data between vaccinated and non-vaccinated mice. One-way ANOVA was used to compare percentages and cell numbers. *P ≤ 0.05, **P ≤ 0.01, ***P ≤ 0.001, ****P ≤ 0.0001.

We further evaluated the expression levels of immunosuppressive factors IL-10 and PD-L1 in CD11b^+^Ly6G^+^ granulocytes and CD11b^+^Ly6C^+^ monocytes in the lungs of vaccinated and non-vaccinated C57BL/6 mice at days 7 and 11 post-intratracheal challenge with a potentially lethal dose of spores isolated from the virulent isolate *Cp* C735, using a regimen outlined in Fig. 3A. We did not attempt to collect data after 11 days post-challenge because some non-protected mice would have approached moribund. ΔT-vaccinated mice maintained their body weight and had significantly reduced fungal burden in the lungs compared to non-vaccinated mice (Fig. 3B and C). These results are in agreement with previously reported data (10, 37). The phenotype and number of lung leukocytes were examined using the same gating strategy for BMMCs shown in Fig. S1. The percentages and numbers of CD11b^+^Ly6G^+^ cells significantly increased in the lungs of non-vaccinated mice compared to vaccinated mice at 7 and 11 DPC (Fig. 3D through F). In contrast, the percentage of CD11b^+^Ly6C^+^ cells in the lungs of non-vaccinated mice showed a significant increase only at 7 DPC compared with vaccinated mice, but this difference was not reflected in the cell number (Fig. 3G and H). Furthermore, the percentage and number of PD-L1^+^ and PD-L1^+^IL-10^+^ double-positive cells were significantly elevated in the CD11b^+^Ly6G^+^ subpopulation of the non-vaccinated mice at 11 DPC, but not in the CD11b^+^Ly6C^+^ subpopulation (Fig. 3I and J). Altogether, these data demonstrated that *Coccidioides*-infected susceptible mice showed elevated numbers of immunosuppressive PD-L1- and IL-10-positive CD11b^+^ Ly6G^+^ cells, characteristic of MDSC-like neutrophils.

### BMMCs exposed to *Cp* inhibited polyclonal CD4^+^ T cell proliferation

**Fig 4:**
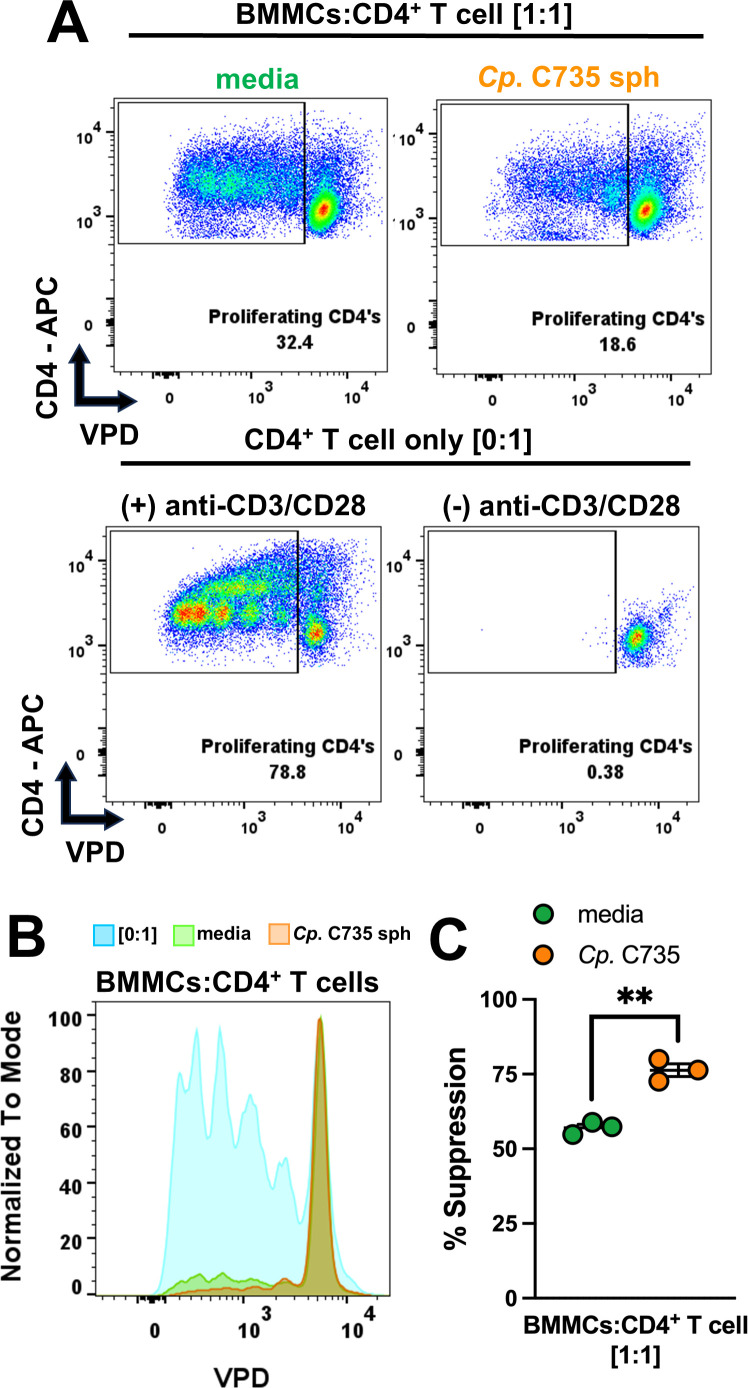
Bone marrow-derived myeloid cells augmented the suppression of CD4^+^ T cell proliferation after exposure to spherules. (**A**) Representative flow cytometry bivariate plots of CD4 (APC labeled) versus violet proliferation dye 450 (VPD450) of gated live single CD4^+^ T cells co-cultured with BMMCs (M:I = 1:1), which were exposed to spherules or media alone. (**B**) Histogram of VPD450 intensity after 72 h and (**C**) percentage of suppression were plotted (*n* = 3 mice; one experiment). Data were analyzed by Student’s *t*-test. **P ≤ 0.01.

We first established a T cell proliferation protocol to examine the function of BMMCs after exposure to *Coccidioides*, as outlined in Fig. S2. Briefly, BMMCs were exposed to PFA-killed spherules prepared from the live-attenuated vaccine strain (ΔT) for 24 h to elicit their function. CD4^+^ T cells were enriched from the spleen of naïve mice and labeled with the violet-proliferation dye 450 (VPD450). The spherule-exposed and control BMMCs were co-cultured with labeled CD4^+^ T cells for 72 h at BMMC:CD4^+^ T cell ratios of 1:1 to 1:16, then analyzed by flow cytometry. Suppression of CD4^+^ T cell proliferation was dose dependent, with the highest suppression at the 1:1 ratio (Fig. S2B through D). The assay condition (BMMCs:CD4^+^ T cells = 1:1) was applied to compare the immunosuppression function of BMMCs in the presence and absence of PFA-killed spherules prepared from the virulent C735 isolate, as shown in Fig. 4A through C. BMMCs exposed to PFA-killed spherules significantly suppressed CD4^+^ T cell proliferation, with an inhibition rate of 76.3%, compared to 57.0% in those cultured with medium alone (Fig. 4C).

**Fig 5:**
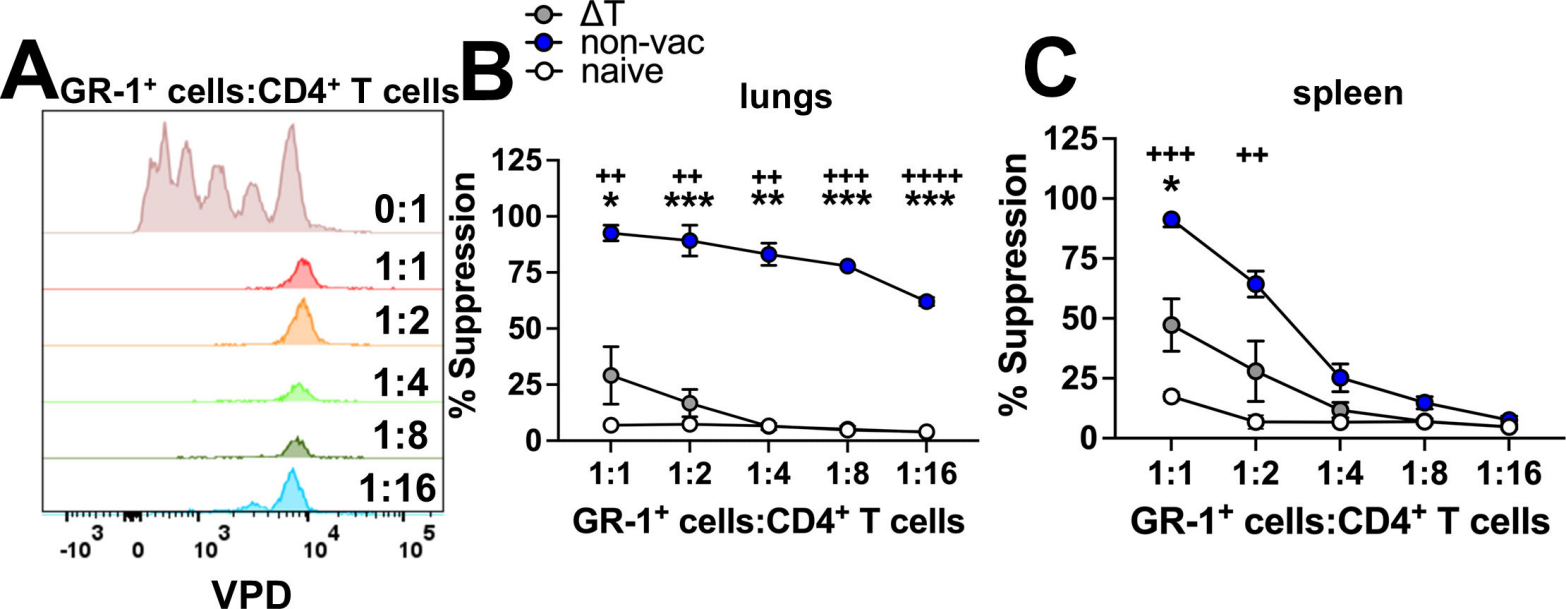
GR-1^+^ granulocytes from the lungs and spleens of *Cp-*infected mice suppressed CD4^+^ T cell proliferation. (**A**) Representative histograms of VPD450 intensity as an indicator of proliferation of CD4^+^ T cells incubated with GR-1^+^ cells isolated from non-vaccinated and *Coccidioides*-infected mice for 72 h at a series of GR-1^+^:CD4^+^ T cell ratios (1:1 to 1:16). Suppression plots (percentages) of CD4^+^ T cell proliferation by GR-1^+^ cells isolated from the lungs (**B**) and the spleen (**C**) of vaccinated (gray dots) versus non-vaccinated (blue dots) mice. GR-1^+^ cells isolated from naïve mice were the negative control (empty circles). *n* = 2–4 mice per group; representative of three experiments. Student’s *t*-test statistically compared data between non-vaccinated and ΔT-vaccinated mice (*) or between non-vaccinated and naïve mice (+). *P ≤ 0.05, **P ≤ 0.01, ***P ≤ 0.001, *^++++^P* ≤ 0.0001, respectively.

We further assessed the inhibition capacity of primary GR-1^+^ cells isolated from the lungs and spleen of vaccinated and non-vaccinated mice that were challenged with *Coccidioides* arthroconidia by the intratracheal route at 11 DPC. At this time, over 83.8% of GR-1^+^ cells isolated from the lungs and spleen of *Coccidioides*-infected mice were Ly6G^+^ (Fig. S3). GR-1^+^ cells were isolated via negative selection; therefore, GR-1^+^ cells were unbound by magnetic beads to prevent activation. Negative selection was used instead of FACS sorting, thereby minimizing cell stress and maintaining cell function and viability. The isolated GR-1^+^ cells were incubated with VPD450-labeled CD4^+^ T cells for 72 h as described above at a series of M:T ratios from 1:1 to 1:16. As expected, enriched primary GR-1^+^ cells from the lungs and the spleen of non-vaccinated mice significantly suppressed the proliferation of CD4^+^ T cells and displayed a dose-dependent response compared to those isolated from the ΔT-vaccinated mice (Fig. 5A through C, blue versus gray circles). Similarly, GR-1^+^ cells isolated from naïve mice (empty circles) displayed low or non-detectable suppression. These data demonstrate that the GR-1^+^ cells recruited to the lungs and spleen during *Cp* infection are predominant MDSC-like cells that suppress CD4^+^ T cell proliferation.

### Adoptive transfer of GR-1^+^ granulocytes isolated from *Coccidioides*-infected mice exacerbated disease

**Fig 6:**
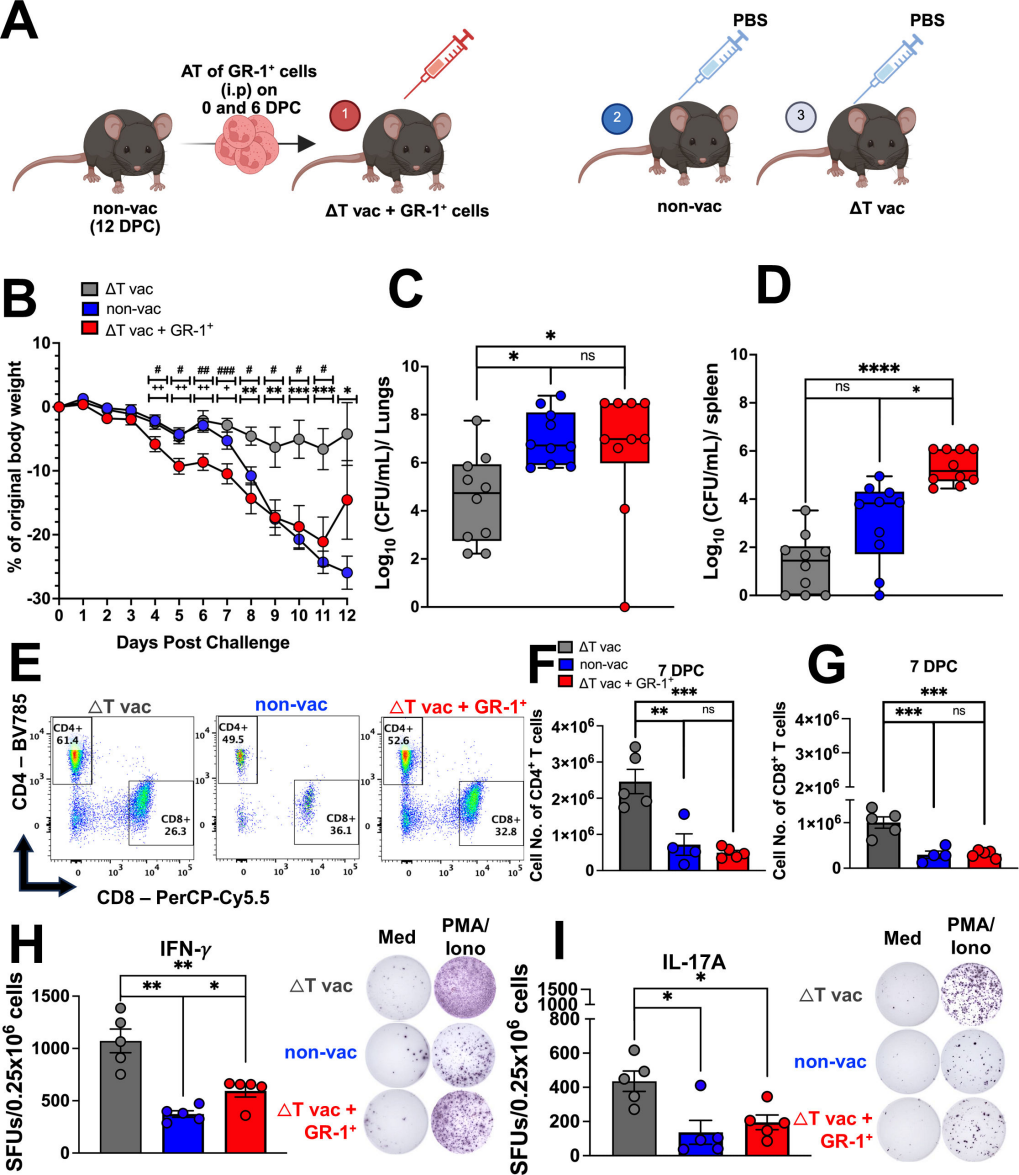
GR-1^+^ granulocytes isolated from *Coccidioides*-infected mice exacerbated disease and reduced protective Th1 and Th17 responses of vaccinated mice. (**A**) A schematic illustration of the adoptive transfer (AT) experiment shows three groups: (1) vaccinated mice that received GR-1^+^ cells, (2) non-vaccinated mice, and (3) vaccinated mice that received PBS as controls. Untouched GR-1^+^ granulocytes were isolated using a negative selection method from non-vaccinated and *Coccidioides*-challenged donor mice at 12 DPC. An aliquot of the isolated cells was analyzed for purity by flow cytometry (≥83%), and 2 × 10^6^ cells per mouse were immediately administered by intraperitoneal injection to the ΔT-vaccinated recipient mice on days 0 and 6 post-challenge. Two control groups received PBS on the same schedule. (**B**) The daily changes in body weight (%) of the three groups of mice were measured and plotted for 12 days. Fungal burden (CFUs on a log_10_ scale) in the lungs (**C**) and spleen (**D**) of these three groups of mice on 12 DPC was plotted. (**E**) Representative bivariate plots of CD4^+^ versus CD8^+^ T cells of gated live single CD45^+^ pulmonary lymphocytes showed a significant reduction of percentages in the lungs of mice receiving GR-1^+^ granulocytes. (**F and G**) Total numbers of CD4^+^ and CD8^+^ T cells in the lungs of each treatment group (*n* = 6), respectively. IFN-γ and IL-17A forming spots (SFUs) were measured using ELISpot assays as described in Materials and Methods and plotted (**H and I**). *n* = 5–10 mice per group; one experiment. *Significant changes in body weight for the ΔT-vaccinated versus non-vaccinated mice; both received PBS; ^+^GR-1^+^ cell recipient mice versus non-vaccinated mice; ^#^GR-1^+^ cell recipient versus ΔT-vaccinated mice. The Kruskal-Wallis test was used to analyze CFU data; numbers of CD4^+^ and CD8^+^ T cells were analyzed with the one-way ANOVA test, and SFUs were compared by Student’s *t*-test. *P ≤ 0.05, **P ≤ 0.01, ***P ≤ 0.001, ****P ≤ 0.0001.

We further investigated whether GR-1^+^ granulocyte cells isolated, via negative selection, from non-vaccinated and *Coccidioides-*infected mice could suppress protective immunity and exacerbate coccidioidomycosis. A group of vaccinated mice (*n* = 10) received 2 × 10^6^ GR-1^+^ cells from non-vaccinated mice by intraperitoneal injection twice: once immediately after an intratracheal *Coccidioides* challenge and again at day 6 post-challenge. Correlates of disease severity were measured using change in body weight and fungal burden as outlined in Fig. 6A. Vaccinated and non-vaccinated mice (*n* = 10) receiving PBS served as controls. Vaccinated mice receiving GR-1^+^ cells (red dots) succumbed to disease and had comparable bodyweight loss to non-vaccinated mice (blue dots). Their body weights showed a significant reduction from days 4 to 11 compared with vaccinated mice receiving PBS alone (Fig. 6B). Furthermore, mice receiving GR-1^+^ cells had a higher fungal burden in the lungs and spleen than vaccinated mice receiving PBS (Fig. 6C and D). Collectively, these data demonstrate that transfer of GR-1^+^ cells from *Cp*-infected mice is sufficient to abrogate vaccine-induced protection against a pulmonary *Cp* challenge. These data suggest that GR-1^+^ cells expressing high levels of PD-L1 play a pathogenic role and exacerbate pulmonary CM.

The GR-1^+^ cell recipient mice showed reduced total cell numbers of CD4^+^ and CD8^+^ T cells compared to the vaccinated mice (Fig. 6E through G). Moreover, there was a significant reduction in IFN-γ- and IL-17A-producing CD4^+^ T cells in the lungs as measured by ELISpot (Fig. 6H and I). These data demonstrate that *Coccidioides*-exposed GR-1^+^ cells play a pathogenic role in *Cp* infection by reducing Th1 and Th17 responses, which have been previously reported to correlate with vaccine-induced protection (10, 38, 39).

**Fig 7:**
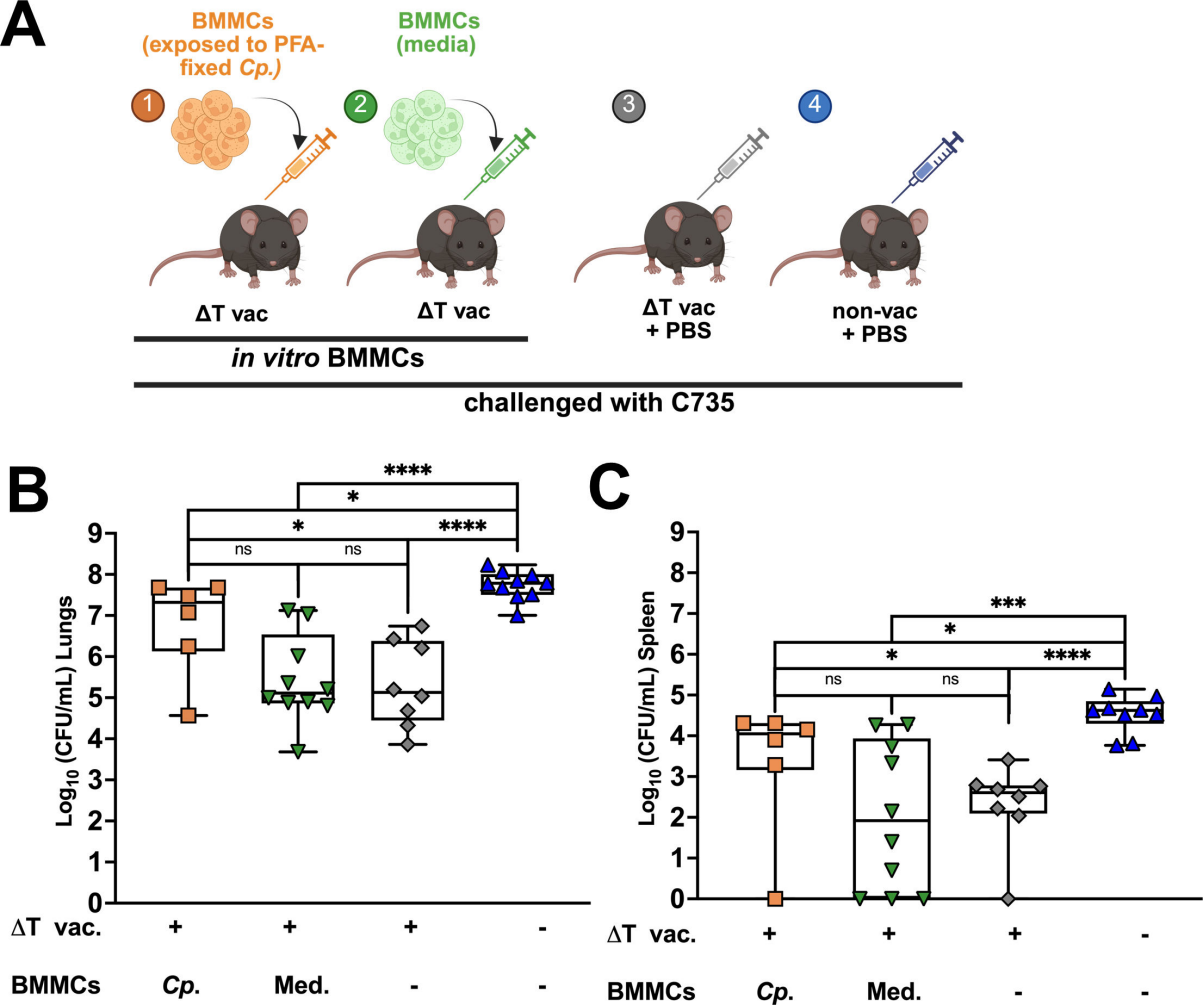
Adoptive transfer of spherule-exposed BMMCs to vaccinated mice exacerbates coccidioidomycosis. (**A**) Bone-marrow cells were cultured as described in Materials and Methods and outlined in Fig. 2A. BMMCs were first primed with PFA-killed spherules (BMMCs: spherules = 5:1) for 24 h and then administered by i.p. injection to ΔT-vaccinated recipient mice (*n* = 6–10, 2 × 10^6^ cells/mouse/time) on days 0, 3, 6, and 9 post-challenge. Three groups of controls, including vaccinated mice receiving untreated BMMCs and vaccinated and non-vaccinated mice receiving PBS, were used for comparison. CFUs on log_10_ scale in the lungs (**B**) and the spleen (**C**) at 12 DPC were plotted. *n* = 6–10 mice per group; one experiment. The Mann-Whitney *U* test was used to compare fungal burden. *P ≤ 0.05,***P ≤ 0001, ****P ≤ 0.0001.

To mitigate the potential transfer of live spherules along with the GR-1^+^ cells from infected mice, we conducted a parallel experiment by adoptively transferring BMMCs exposed to PFA-fixed spherules into ΔT-vaccinated mice. A group of vaccinated mice that received BMMCs cultured in medium was included, along with two control groups of vaccinated and non-vaccinated mice receiving PBS alone (Fig. 7A). Mice were injected with 2 × 10^6^ BMMCs or PBS right after challenge and on days 0, 3, 6, and 9 post-challenge. Data revealed that vaccinated mice that received spherule-primed BMMCs had a significant increase in fungal burden in the lungs and spleen as compared with their vaccinated cohort that received PBS alone (Fig. 7B and C). These data are consistent with the adoptive transfer of primary GR-1^+^ cells isolated from infected mice (Fig. 6) and demonstrated that GR-1^+^ granulocytes exposed to *Cp* differentiated into MDSC-like cells, which suppress protective immunity, reduce fungal clearance, and exacerbate disease in vaccinated mice.

## DISCUSSION

Both laboratory and clinical studies have demonstrated that the onset of coccidioidomycosis is associated with elevated circulating neutrophils, which can infiltrate pulmonary lesions (15, 40–43). Human neutrophils exhibit strong chemotactic, adhesive, and phagocytic responses to endospores and young spherules, but are unable to engulf mature spherules due to their large size (44). While neutrophils readily phagocytose coccidioidal arthroconidia, young spherules, and endospores, killing is inefficient, and they may survive within host cells (14, 45). Likewise, remarkably high numbers of CD11b^+^Ly6G^+^ cells were detected in the lungs of *Coccidioides*-infected mice, and these cells were associated with disease outcome (10, 46). Though neutrophil phagocytic killing has been studied, little is known about their immunological modulation roles during coccidioidomycosis. Here, we show that exposure to *Coccidioides* induces PD-L1 expression on CD11b^+^Ly6G^+^ neutrophils, which are functionally similar to MDSCs. Additionally, we demonstrate that recruited GR-1^+^ granulocytes to the lungs and spleen of infected mice, composed of PD-L1-expressing cells, can suppress vaccine-induced protective Th1 and Th17 memory responses, thereby exacerbating disease. While *C. posadasii* and *C. immitis* infections produce a wide spectrum of clinical manifestations, certain clinical isolates exhibit attenuated virulence in murine models. We demonstrated the immunosuppressive capacity of MDSC-like neutrophils during infection with the highly virulent *C. posadasii* isolate C735 in this study; however, the precise role of these cells in the host response to attenuated *Coccidioides* strains remains to be elucidated.

MDSCs play a detrimental role during pulmonary coccidioidomycosis in mice. The infiltrated Ly6G^+^ neutrophils in the lungs express elevated levels of PD-L1 and function as MDSCs. Depending on the immunological and pathological context, MDSCs can play both detrimental and beneficial roles. *C. dubliniensis* infection induces MDSCs as part of a trained innate response that protects against subsequent infection with *C. albicans* and *Staphylococcus aureus*, which cause harmful inflammation and lethal sepsis (28, 29). In this context, MDSCs benefit the host by suppressing detrimental inflammation (28, 29). On the contrary, *Cryptococcus neoformans* and *Paracoccidioides brasiliensis* infections recruit Ly6G^+^ MDSCs to the lungs, which worsen the diseases by suppressing protective immune responses like Th1 and Th17 immunity (32, 47). In this study, we revealed that *Coccidioides* infection recruits MDSC-like neutrophils and exacerbates the disease in non-protected mice. Importantly, vaccination can reduce the infiltration of these immunosuppressive cells into the lungs infected with *Coccidioides*.

MDSCs share many lineage markers (e.g., CD11b, GR-1, Ly6G, and Ly6C) with other granulocytes and monocytes; thus, functional assays are essential to characterize the role of MDSCs in microbial infections. We demonstrate that BMMCs exposed to *Cp* spherules and arthroconidia *in vitro* differentiate into CD11b^+^Ly6G^+^ or CD11b^+^Ly6C^+^ cells, both of which suppress the proliferation of CD4^+^ T cells in a dose-dependent manner. Similarly, we have revealed that GR-1^+^ cells isolated from the lungs and spleen of non-protected mice expressed elevated levels of PD-L1 and significantly suppressed CD4^+^ T cells to a greater extent than GR-1^+^ cells enriched from vaccine-protected and naïve mice. Furthermore, adoptive transfer of GR-1^+^ cells isolated from the lungs of diseased mice into vaccine-protected mice validates the immunosuppressive function of the recruited myeloid cells. Likewise, transfer of spherule-exposed BMMCs to vaccine-protective recipients resulted in higher fungal burden in the lungs. In contrast, this effect was not observed in recipient mice that received BMMCs cultured in media alone. The increase of fungal burden is associated with elevated PD-L1 expression on myeloid cells after *Cp* exposure. *In vivo*, we detected a consistent increase in PD-L1 expression on Ly6G^+^ cells but not on Ly6C^+^ cells after *Coccidioides* challenge. Altogether, these data reveal the role of CD11b^+^PD-L1^+^Ly6G^+^ neutrophils in suppressing protective T cell responses during pulmonary *Coccidioides* infection.

MDSCs are able to suppress T cell responses through a variety of mechanisms, including the induction of anergy through PD-L1 engagement with PD-1 on T cells, the secretion of IL-10 to inhibit the activation of other immune cells, the expression of an elevated amount of 2,3-dioxygenase 1 to deplete tryptophan, an amino acid vital for T cell proliferation, and the production of NO contributing to the suppression of T cell function (48, 49). Indeed, we have found that Ly6G^+^ MDSC-like neutrophils in the lungs of diseased mice upregulate PD-L1 and IL-10. We have demonstrated, for the first time, that upregulation of PD-L1 on Ly6G^+^ cells is associated with disease severity through the adoptive transfer of granulocytes isolated from *Coccidioides* infection. A similar report has shown that a population of neutrophils expressing PD-L1 is recruited to the lungs of *Coccidioides*-infected mice using single-cell RNA-seq analysis, while no functional assay was used to validate those data (35, 36). PD-L1 binds to its receptor, PD-1, on T cells, sending signals that suppress T cell function. It is conceivable that upregulation of PD-L1 could suppress the protective T cell response against *Coccidioides* infection. Further exploration of PD-L1 function using loss- and gain-of-function mutations or PD-L1 inhibitors would further validate the immunosuppressive effect of the PD-L1-PD-1 axis during *Coccidioides* infection.

Extensive evidence supports the notion that persistently elevated IL-10 production at the onset of coccidioidomycosis contributes to pathogen proliferation, dissemination, and disease severity (50–52). A previous investigation of IL-10-producing cells during *Coccidioides* infection revealed that IL-10 is upregulated in CD8^+^ T cells and Ly6G^+^ neutrophils in the lungs of non-protective mice. In contrast, CD4^+^ T cells, DCs, and macrophages are the predominant producers of IL-10 in vaccinated mice, although the total IL-10 concentration is much lower in vaccinated mice (17). Our current data agree with the previous finding that Ly6G^+^ myeloid cells are the primary source of IL-10 in mice with severe coccidioidomycosis. In a recent study, Li et al. demonstrated that PD-L1 directly binds to the yeast ribosomal protein RPL20B, thereby modulating IL-10 production in bone marrow-derived macrophages (53). This article suggests that macrophages play an immunosuppressive role in yeast infection; however, direct evidence is needed to validate this function. We discovered that neutrophils, not macrophages, are the major innate cells recruited to the infection sites and upregulate both PD-L1 and IL-10 production. It remains unknown whether any *Coccidioides* protein can bind PD-L1 to regulate IL-10 production in macrophages to suppress host immunity, and this requires further study.

These data imply that depleting MDSCs and/or inhibiting the PD-L1/PD-1 axis are potential therapeutic strategies to enhance host immunity against *Coccidioides*. Our laboratory has previously shown that vaccine-protected mice display early recruitment of Ly6G^+^ neutrophils to a moderate level. Depletion of these cells with a specific anti-Ly6G monoclonal antibody (clone 1A8) renders partial protective efficacy (10, 11). These results suggest that Ly6G^+^ cells are essential for protective immunity against *Coccidioides* infection. However, a sharp increase in Ly6G^+^ neutrophils occurs in non-protected mice after 7 days post-challenge with *Coccidioides*, reaching an extremely high level (83% of leukocytes in the lungs) as they approach moribundity. It is plausible that the early recruited Ly6G^+^ cells differentiate into MDSC-like neutrophils when exposed to spherules in the diseased mice. Therefore, depletion of Ly6G^+^ cells early after infection may benefit disease outcomes. This strategy has been successfully demonstrated for *Paracoccidioides* infection, where depletion of MDSCs with anti-GR-1 antibodies and 5-fluorouracil has been shown to improve tissue pathology and elevate protective immune response (47, 54). Similarly, blocking PD-1 has improved disease outcome in murine models of paracoccidioidomycosis, pulmonary mucormycosis, and invasive pulmonary aspergillosis (55–57). Our results support the notion that immunotherapy targeting MDSC-like neutrophils and/or the PD-L1/PD-1 axis may benefit the host in terms of disease outcome in coccidioidomycosis.

## MATERIALS AND METHODS

### Fungal strain, growth conditions, and spore preparation

PFA-killed spores and spherules were prepared from a virulent clinical isolate of *Coccidioides posadasii* C735 and an attenuated vaccine strain (ΔT) derived from this isolate (37). The saprobic phase was grown on GYE agar (1% glucose, 0.5% yeast extract, 1.5% agar) to produce spores as previously reported (58). The pulmonary challenge inoculum (spores) was prepared from the C735 isolate. Spherules were grown in Converse medium, a chemically defined medium, for 4–5 days in a shaking incubator at 10% CO_2_ and 39°C. All culturing and preparatory procedures of *C. posadasii* were conducted in a biosafety level 3 laboratory located at the University of Texas at San Antonio (UTSA).

### Mice, vaccination protocol, pulmonary challenge, and evaluation of protection

Six- to eight-week-old female C57BL/6 mice were purchased from Charles River Laboratories. C57BL/6 mice were subcutaneously immunized with an attenuated vaccine (ΔT) in the abdominal region at 2-week intervals, with 5 × 10^5^ spores and then 2.5 × 10^4^ spores, as reported previously (37). Mice were challenged intratracheally with a suspension of 50–115 viable spores (CFUs) of *C. posadasii* in 50 μL of DPBS at 4 weeks after the final vaccination, as reported previously (58). Mice were euthanized at the indicated days post-challenge for the determination of fungal burden in the lungs and the spleen, and immune cell profiling by flow cytometry, as previously described (58).

### Flow cytometry analysis and ELISpot

Single-cell suspensions were prepared from the lungs and spleens of treated and control mice at the indicated time points, as reported previously (39, 58). Standard flow cytometry methodology was used for direct monoclonal antibody labeling and enumeration of pulmonary and splenic leukocytes using a BD LSRII cytometer. Leukocytes were stained with a cocktail of directly labeled antibodies to detect myeloid cell populations: FITC-anti-mouse CD45 (clone 30-F11; BioLegend,103108), BV785 anti-mouse/human CD11b (clone M1/70; BioLegend, 101243), PerCP-Cy5.5 anti-mouse Ly6G (clone 1A8; BioLegend, 127616), PE anti-mouse Ly6C (clone AL-21; BD, 560592), R718 anti-mouse PD-L1 (clone MIH5; BD, 567584), and BV421 anti-mouse IL-10 (clone JES5-16E3; BioLegend, 505022). Leukocytes were permeabilized with a BD Cytofix/Cytoperm Kit and stained with a cocktail of fluorochrome-conjugated detecting antibodies: BV421 anti-mouse IFN-γ (clone XMG1.2; BD, 563376), PE anti-mouse IL-17A (clone TC11-18H10.1; BioLegend, 506904), BV785 antimouse CD4 (clone GK1.5; BioLegend, 100453), FITC anti-mouse TCR β chain (clone H57-597; BioLegend, 109206), and PerCP-Cy5.5 anti-mouse CD8b molecules (clone YTS156.7.7; BioLegend, 126610). Data were analyzed using FlowJo software version 10.9. IL-17A and IFN-γ-producing CD4^+^ T cells in the lungs and spleen were evaluated using ELISpot assays, as reported previously (58, 59).

### *In vitro* BMMCs

The tibia and femur were collected and flushed using a 26-G needle with 10 mL of RPMI 1640 supplemented with 10% FBS (Gibco), 100 U/mL of penicillin, and 100 μg/mL of streptomycin (Gibco), 1× GlutaMAX (Gibco), and 1× β-mercaptoethanol (Gibco) and passed through a 70 μm filter. Red blood cells were lysed using ACK lysis buffer (Gibco) according to the manufacturer’s instructions. In brief, BM cells were cultured at 1 × 10^6^ cells per well in a 24-well plate with 40 ng/mL of GM-CSF (R&D Systems). On day 3, cells were supplemented with an additional 40 ng/mL of GM-CSF, and PFA-fixed spherules and arthroconidia were added at a 5:1 ratio (mammalian cells:*Coccidioides* cells) (60, 61).

### Confocal imaging

Bone marrow-derived myeloid cells were seeded in 18-well chamber slides (µ-Slides, Ibidi) at 5 × 10^5^ cells/mL in PBS and allowed to settle for 35 min at room temperature. Cells were fixed with 100 μL of 2% paraformaldehyde in the chamber for 10 min at room temperature. The chamber was washed three times with 100 μL PBS, then incubated for 10 min with 100 μL 0.5% Triton X-100 to permeabilize cells. The cells were washed three times and blocked with 2% BSA for 48 h at 4°C. Hoechst staining was conducted as described in the manufacturer’s protocol (Thermo Fisher).

### Isolation of GR-1^+^ cells and functional assays

GR-1^+^ cells were isolated from the lungs and spleen of the indicated mouse groups and passed through a 70 μm filter, as reported previously (39, 58). Briefly, GR-1^+^ (Ly6G^+^ and Ly6C^+^) cells were enriched using a STEMCELL negative selection magnetic bead kit. Thus, GR-1^+^ cells remained unbound by magnetic beads to prevent activation and were resuspended in cRPMI. CD4^+^ T cells were enriched from naive mice using a STEMCELL negative selection magnetic bead kit and stained with violet-proliferation dye (VPD) to track cell divisions. After VPD staining, T cells were added to a 96-well plate containing anti-CD3/CD28 Dynabeads (Thermo Fisher) at 5 × 10^4^ cells per well, and GR-1^+^ cells were serially diluted and plated at ratios of 1:1, 1:2, 1:4, 1:8, and 1:16 (GR-1^+^ cells:CD4^+^ T cells), as described previously (62). Proliferation was analyzed using FlowJo version 10.9.

### Statistical analyses

Student’s *t*-test was used to analyze percentages and cell counts of infiltrated immune cells in the lungs and spleen between ΔT-vaccinated and non-vaccinated groups. One-way ANOVA was used to compare cell numbers and ELISpot results across ≥3 independently treated groups. When comparing fungal burden, the Mann-Whitney test, a non-parametric method for two groups, or the Kruskal-Wallis test for ≥3 independently treated groups was used, as previously reported (10). A *P* value of equal to or less than 0.05 was considered statistically significant (38, 58).

## Data Availability

The data underlying this article will be shared upon reasonable request to the corresponding author.

## SUPPLEMENTAL MATERIAL

Figure S1 mbio.00772-26-s0001.pdf Gating strategy of CD11b^+^Ly6G^+^ and Ly6C^+^ cells differentiated from bone marrow with GM-CSF.

Figure S2 mbio.00772-26-s0002.pdf Bone marrow-derived myeloid cells exposed to an attenuated strain of *Cp* inhibit polyclonal CD4^+^ T cell proliferation.

Figure S3 mbio.00772-26-s0003.pdf Purity of *ex vivo* pulmonary GR-1^+^ cells.

ASM does not own the copyrights to Supplemental Material that may be linked to, or accessed through, an article. The authors have granted ASM a non-exclusive, world-wide license to publish the Supplemental Material files. Please contact the corresponding author directly for reuse.
