# Supplementary material for: Myeloid-derived suppressor cell (MDSC)-like neutrophils induced by pulmonary infection with Coccidioides posadasii exacerbate disease by suppressing CD4+ T cell immunity
Source: mBio. 2026 May 28;17(7):e00772-26. doi: 10.1128/mbio.00772-26 (PMC13343843; doi:10.1128/mbio.00772-26)
Supplement: Figure S1 — Gating strategy of CD11b+Ly6G+ and Ly6C+ cells differentiated from bone marrow with GM-CSF. [file mbio.00772-26-s0001.pdf]

Supplemental Figure 1

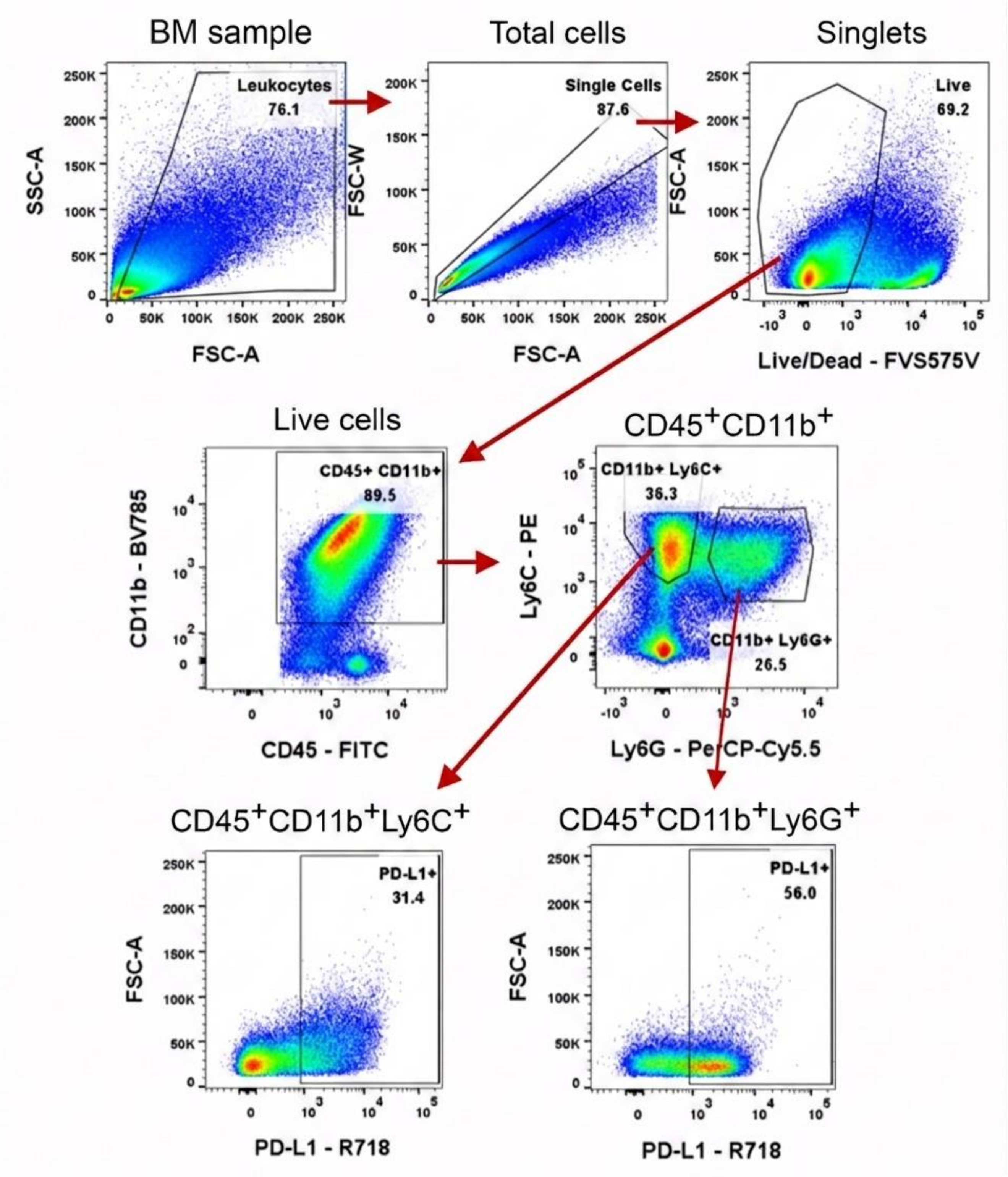

Supplemental Figure 1. Gating Strategy of $CD11b^{+}$ $Ly6G^{+}$ and $Ly6C^{+}$ cells differentiated from bone marrow with GM-CSF. Total cell, singlet, and live cell gates were used to excluded debris, doublets and dead cells from analysis, respectively. Live cells were then gated for $CD45^{+}$ $CD11b^{+}$ cells, which were subsequently separated into $Ly6C^{+}$ and $Ly6G^{+}$ cell population. $Ly6G^{+}$ cells and $Ly6C^{+}$ cells were separately gated on to observe PD-L1 expression.
