# Supplementary material for: Myeloid-derived suppressor cell (MDSC)-like neutrophils induced by pulmonary infection with Coccidioides posadasii exacerbate disease by suppressing CD4+ T cell immunity
Source: mBio. 2026 May 28;17(7):e00772-26. doi: 10.1128/mbio.00772-26 (PMC13343843; doi:10.1128/mbio.00772-26)
Supplement: Figure S2 — Bone marrow-derived myeloid cells exposed to an attenuated strain of Cp inhibit polyclonal CD4+ T cell proliferation. [file mbio.00772-26-s0002.pdf]

Supplemental Figure 2.

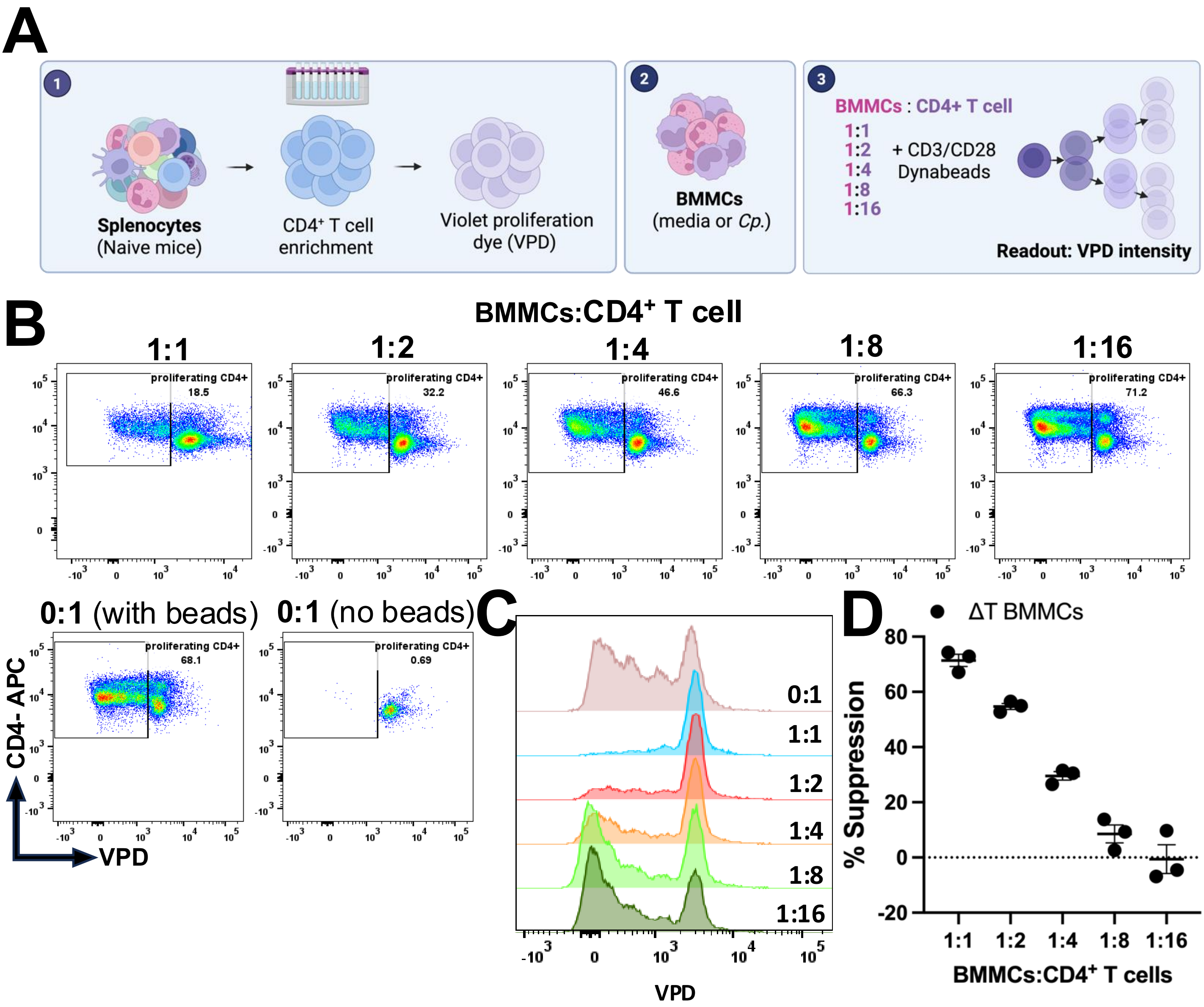

Supplemental Figure 2. Bone-marrow derived myeloid cells exposed to an attenuated strain of *Cp.* inhibit polyclonal CD4⁺ T cell proliferation

(A). In a functional assay, differentiated BM cells and VPD-labeled $CD4^+$ T cells were cultured with anti-CD3/CD28 beads for 72 hours and $CD4^+$ T cell proliferation was analyzed (B). Flow cytometric analysis of VPD450-labeled $CD4^+$ T cells (C). Histogram of VPD450 demonstrating the division of VPD450-labeled $CD4^+$ T cells. (D). Suppression of $CD4^+$ T cell proliferation (n=3)
