# Supplementary material for: Myeloid-derived suppressor cell (MDSC)-like neutrophils induced by pulmonary infection with Coccidioides posadasii exacerbate disease by suppressing CD4+ T cell immunity
Source: mBio. 2026 May 28;17(7):e00772-26. doi: 10.1128/mbio.00772-26 (PMC13343843; doi:10.1128/mbio.00772-26)
Supplement: Figure S3 — Purity of ex vivo pulmonary GR-1+ cells. [file mbio.00772-26-s0003.pdf]

Supplemental Figure 3

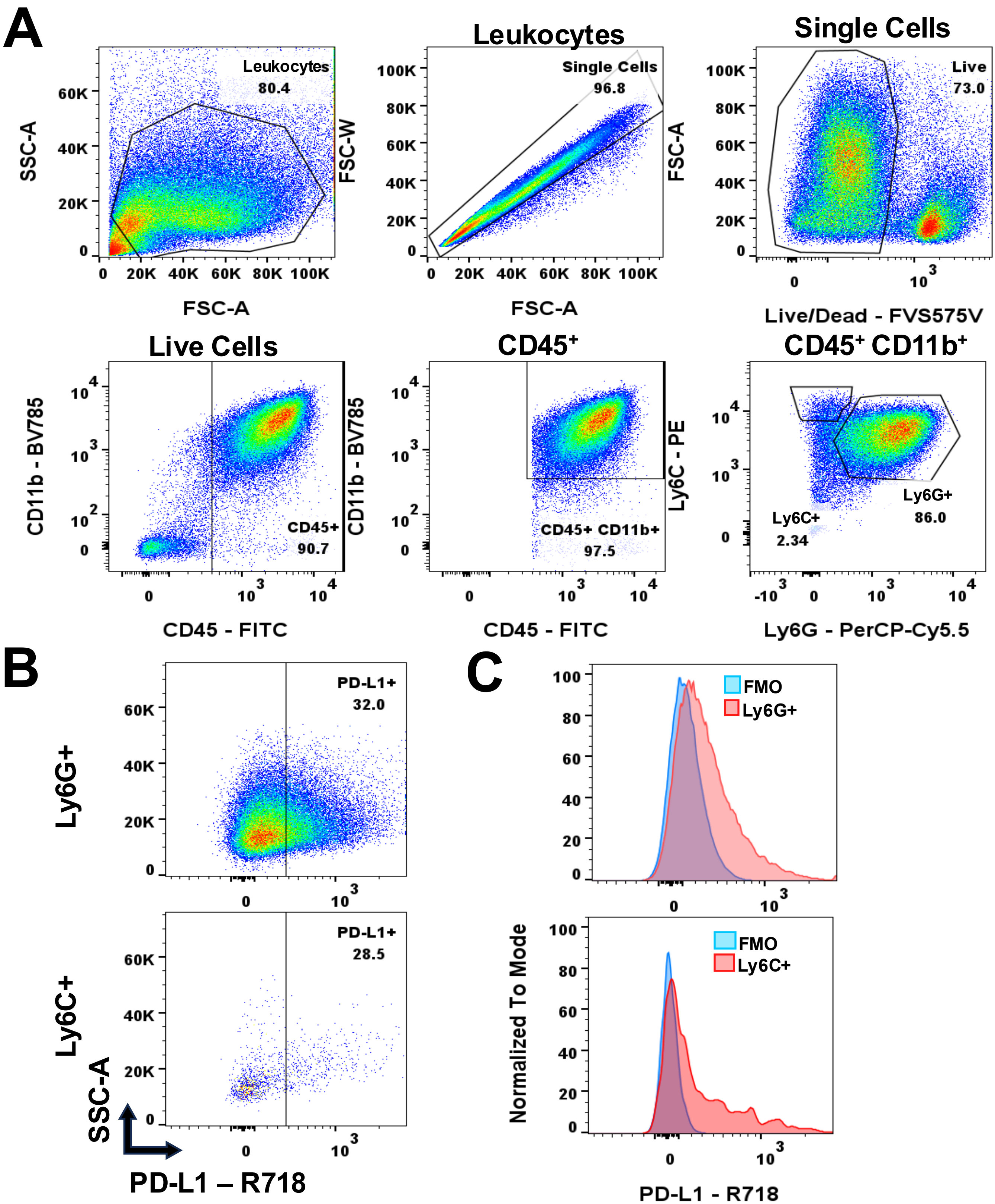

Supplemental Figure 3. Purity of *ex vivo* pulmonary GR-1^{+} cells

(A). Gating strategy for CD11b^{+} Ly6G^{+} and CD11b^{+} Ly6C^{+} cells 12 DPC after enrichment using the GR-1 negative selection kit (STEMCELL) (B). Flow cytometric analysis of PD-L1 expression on Ly6G^{+} or Ly6C^{+} cells C). Histogram demonstrating PD-L1 MFI
